# Probabilistic modeling of bifurcations in single-cell gene expression data using a Bayesian mixture of factor analyzers

**DOI:** 10.12688/wellcomeopenres.11087.1

**Published:** 2017-03-15

**Authors:** Kieran R Campbell, Christopher Yau

**Affiliations:** 1 Department of Physiology, Anatomy and Genetics, University of Oxford, Oxford, OX1 3QX, UK; 2 Wellcome Trust Centre for Human Genetics, University of Oxford, Oxford, OX3 7BN, UK; 3 Centre for Computational Biology, Institute of Cancer and Genomic Sciences, University of Birmingham, Birmingham, B15 2TT, UK

**Keywords:** Single-cell, gene expression, bifurcation, Bayesian, factor analysis

## Abstract

Modeling bifurcations in single-cell transcriptomics data has become an increasingly popular field of research. Several methods have been proposed to infer bifurcation structure from such data, but all rely on heuristic non-probabilistic inference. Here we propose the first generative, fully probabilistic model for such inference based on a Bayesian hierarchical mixture of factor analyzers. Our model exhibits competitive performance on large datasets despite implementing full Markov-Chain Monte Carlo sampling, and its unique hierarchical prior structure enables automatic determination of genes driving the bifurcation process. We additionally propose an Empirical-Bayes like extension that deals with the high levels of zero-inflation in single-cell RNA-seq data and quantify when such models are useful. We apply or model to both real and simulated single-cell gene expression data and compare the results to existing pseudotime methods. Finally, we discuss both the merits and weaknesses of such a unified, probabilistic approach in the context practical bioinformatics analyses.

## Introduction

Trajectory analysis of single-cell RNA-seq (scRNA-seq) data has become a popular method that attempts to infer lost temporal information, such as a cell’s differentiation state ^1, 2^. Such analyses reconstruct a measure of a cell’s progression through some biological process, known as a *pseudotime*. Recently, attention has turned to modeling bifurcations where, part-way along such trajectories, cells undergo some fate decision and branch into two or more distinct cell types.

Several methods have been proposed to infer bifurcation structure from single-cell data. Wishbone ^3^ constructs a *k*-nearest neighbor graph and uses shortest paths from a *root* cell to define pseudotimes, using inconsistencies over multiple paths to detect bifurcations. Diffusion Pseudotime (DPT) ^4^ similarly constructs a transition matrix where each entry may be interpreted as a diffusion distance between two cells. Bifurcations are inferred by identifying the anti-correlation structure of random walks from both a root cell and its maximally distant cell. While DPT arguably has a probabilistic interpretation, neither method specifies a fully generative model that incorporates measurement noise, while both infer bifurcations retrospectively after constructing pseudotimes. A further algorithm Monocle ^5^ learns pseudotimes based on dimensionality reduction using the DDRTree algorithm ^6^ and provides post-hoc inference of genes involved in the bifurcation process using generalized linear models.

Here we propose a Bayesian hierarchical mixture of factor analyzers for inferring bifurcations from single-cell data. Factor analysis and its close relative principal component analysis (PCA) are frequently used in the context of single-cell gene expression modeling, both for visualization and trajectory inference (see e.g. 7, 8). Since developmental bifurcations involve two related processes, it is therefore natural to extend such models to involve a mixture of two factor analyzers in a Bayesian hierarchical setting that relates expression patterns between branches.

The model we propose is unique compared to existing bifurcation inference methods methods in the following: (1) by specifying a fully generative probabilistic model we incorporate measurement noise into inference and provide full uncertainty estimates for all parameters; (2) we simultaneously infer cell “pseudotimes” and branching structure as opposed to post-hoc branching inference as is typically performed; and (3) our hierarchical shrinkage prior structure automatically detects features involved in the bifurcation, providing statistical support for detecting which genes drive fate decisions.

In the following, we introduce our model and apply it to both synthetic datasets and demonstrate its consistency with existing algorithms on real single-cell data. We further propose a zero-inflated variant that takes into account zero-inflation, and quantify the levels of dropout at which such models are beneficial. We highlight the multiple natural solutions to bifurcation inference when using gene expression data alone and finally discuss both the merits and drawbacks of using such a unified probabilistic model.

## Methods

### Statistical model

We begin with an *N × G* matrix of suitably normalized gene expression measurements for *N* cells and *G* genes, where **y** *_i_* denotes the *i ^th^* row vector corresponding to the expression measurement of cell *i*. We assign a pseudotime *t _i_* to each cell, along with a binary variable *γ* *_i_* indicating to which of *B* branches cell *i* belongs:

*γ* *_i_* = *b* if cell *i* on branch *b*                                      (1)

with *b* ∈ 1,…, *B*.

The pseudotime *t*_*i*_ is a surrogate measure of a cell’s progression along a trajectory while it is the behavior of the genes - given by the factor loading matrix - that changes between the branches. We therefore introduce *B* factor loading matrices Λ *_b_* = [ **c** *_b_* **k** *_b_*], *b* ∈ 1,…, *B* for each branch modeled.

The likelihood of a given cell’s gene expression measurement conditional on all the parameters is then given by

**y** *_i_*| *γ* *_i_*,Λ _*γ_i_*_, *t _i_*, ***τ*** ∼ Normal( **c**_*γ_i_*_ + **k**_*γ_i_*_ *t _i_*, **τ** ^–1^ *_G_*)                        (2)

where _*G*_ is the *G × G* identity matrix.

We motivate the prior structure as follows: if the bifurcation processes share some common elements then the behavior of a non-negligible subset of the genes will be (near) identical across branches. It is therefore reasonable that the factor loading gradients **k**_*γ*_ should be similar to each other unless the data suggests otherwise. We therefore place a prior of the form

**k**_*γ*_i__ ∼ Normal( ***θ***, ***χ*** ^–1^ *_G_*)                                         (3)

where ***θ*** denotes a common factor gradient across branches. This has similar elements to Automatic Relevance Determination (ARD) models with the difference that rather than shrinking regression coefficients to zero to induce sparsity, we shrink factor loading gradients towards a common value to induce similar behavior between mixture components. We can then inspect the posterior precision to identify genes involved in the bifurcation: if *χ _g_* is very large then the model is sure that *k*_0 *g*_ ≈ *k*_1 *g*_ and gene *g* is not involved in the bifurcation; however, if *χ _g_* is relatively small then | *k*_0 *g*_ – *k*_1 *g*_| >> 0 and the model indicates that *g* is involved in the bifurcation.

With these considerations the overall model is given by the following hierarchical (M)ixtures of (F)actor (A)nalysers (MFA) specification:

$$\begin{array}{l} \;\omega ∼\text{Dirichlet}(1/B,\ldots ,1/B)\\ \;\gamma_{i}∼\text{Categorical}(\omega )\\ \;\eta ∼\text{Normal}(\tilde{\eta },\tau_{\eta }^{–1})\\ \theta_{g}∼\text{Normal}(\tilde{\theta },\tau_{\theta }^{–1})\\ \chi_{g}∼\text{Gamma}(\alpha_{x},\beta_{x})\;(4)\\ {\text{c}}_{\gamma_{i}}∼\text{Normal}(\eta ,\tau_{c}^{–1})\\ {\text{k}}_{\gamma_{i}}∼\text{Normal}(\theta ,\chi^{–1}{}_{G})\\ \;t_{i}∼\text{Normal}(0,1)\\ \;\text{τ}∼\text{Gamma}(\alpha ,\beta )\\ \;\;{\text{y}}_{i}∼\text{Normal}({\text{c}}_{\gamma_{i}}+{\text{k}}_{\gamma_{i}}t_{i},\tau^{–1}{}_{G}) \end{array}$$

where $\tilde{\eta },\tilde{\theta }$, *τ _η_*, *τ _θ_*, *τ _c_*, *α _χ_*, *β _χ_*, *α* and *β* are hyperparameters fixed by the user. By default we set the non-informative prior *α _χ_* = *β _χ_* = 10 ^−2^ to maximize how informative the posterior of ***χ*** is in identifying genes that show differential expression across the branches.

As the model exhibits complete conditional conjugacy, inference was performed using Gibbs sampling ( Supplementary File 1). Details of computer software (MFA) implementing these methods is given in Software availability ^9^.

### Modeling zero-inflation

Single-cell data is known to exhibit *dropout* where the failure to reverse-transcribe lowly expressed mRNA results in zero counts in the expression matrix. The issue has been extensively studied in the context of scRNA-seq, resulting in algorithms that take into account the resulting zero inflation, such as ZIFA ^7^ or SCDE ^10^.

We can incorporate tractable zero-inflation into our model by considering a per-gene dropout probability given by

$$p(\text{dropout in gene}g)=\text{exp}\left(-\frac{\lambda }{N}\sum_{i=1}^{N}x_{ig}\right)\;(5)$$

where *x _ig_* is the unobserved true expression of gene *g* in cell *i* and *λ* is a global dropout parameter estimated in an Empirical-Bayes manner. This exponential model empirically fits multiple scRNA-seq datasets well ( Supplementary File 1). Incorporating this zero-inflated likelihood modifies the model in 4 to

$$\begin{array}{l} \;{\text{x}}_{i}∼\text{Normal}\left({\text{c}}_{\gamma_{i}}+{\text{k}}_{\gamma_{i}}t_{i},\tau^{–1}{}_{G}\right)\\ h_{ig}∼\text{Bernoulli}(\text{exp}\left(–\frac{\lambda }{N}\sum_{i}^{}x_{ig}\right))\;\left(6\right)\\ {\text{y}}_{ig}=\{\begin{array}{cc} x_{ig} & \mathrm{if}h_{ig}=0\\ 0 & \text{if}\;h_{ig}=1 \end{array} \end{array}$$

While incorporating zero-inflation in the likelihood leads to a less-misspecified model, we must perform inference on an additional *N*_0_ parameters, where *N*_0_ is the number of zero measurements in the expression matrix. For single cell RNA-seq data this can be as high as 90% of all measurements, leading to a significant additional computational burden.

Furthermore, such a dropout model assumes a per-gene dropout probability dependent on the mean latent expression, though in reality the dropout probability would depend on the latent expression itself. This compromise allows us to estimate the parameter *λ* by fitting for each gene the proportion of cells expressed versus the mean expression.

### Multiple solutions to bifurcation inference

It is common in bifurcation inference methods to specify additional information aside to gene expression data alone. For example, Wishbone requires the specification of a *root* cell that signifies the beginning of pseudotime. DPT also allows for the specification of a root cell or picks the furthest from a random cell if unspecified. Monocle equivalently allows re-fitting of the pseudotimes with the constraint that one of the inferred “states” is the initial or root state.

**Figure 1.**
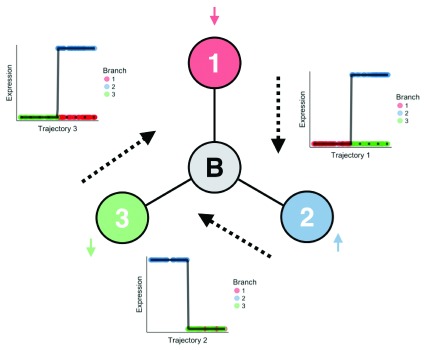
Multiple solutions to bifurcation inference. Starting with three cell states, we would like to infer a bifurcation process from one to the other two. If a single gene is up-regulated in one of the states, yet down-regulated in the other two, then clearly any state may act as the beginning of the trajectory. For example, if we start in state 1 then the gene is up-regulated along state 2 and stays constant in state 3; if we start in state 2 then the gene is down-regulated in states 1 & 3; if we start in state 3 then the gene is up-regulated in state 2 and remains down-regulated in state 1. However, due to the non-identifiability this is true if we add additional genes that are up-regulated in one or two of the cell states. The equivalent geometric argument is that we can build the transcriptomic profiles across all genes by spinning the figure about B (with possible inversion) and “adding” that gene. No matter how many additional genes we add, any one of the three states can act as the root state or beginning of pseudotime. Therefore, in the absence of any additional information there are always three equally valid solutions to bifurcation inference from gene expression data alone.

We argue that such requirements are necessary due to a fundamental invariance in the gene expression of bifurcating cells. Figure 1 shows a conceptual model of three end-states (1–3) and a gene that is expressed in one end state (2), but not the others. We can envisage three possible bifurcation routes here: state 1 is the initial state that bifurcates to 2 & 3 (1 → 2, 3), or equivalently 3 → 1, 2 or 2 → 1, 3. If 1 or 3 is the initial state then the gene exhibits differential expression across the branches, while if we start at 2 the gene exhibits concordant expression across the branches. Note that for a bifurcation we require some genes that show differential expression between the branches and some that show concordant expression - lacking the former would give a non-branching trajectory and lacking the latter would give separate cell types.

The above reasons that in a single-gene case the initial state is indistinguishable from the gene expression alone. We can easily generalize this to the multiple-gene case, due to the fact that the labels in Figure 1 are statistically non-identifiable. The equivalent geometric argument is that you can ‘spin’ Figure 1 about **B** for each gene (and optionally invert the expression to give two states of non-zero expression).

While in algorithms, such as Wishbone and DPT, this non-identifiability is solved by setting an initial cell or state, the equivalent in our model is the correct initialization of the pseudotimes. PCA is applied to the data before inference and the principal component that best corresponds to the trajectory based on the expression of known genes is used to initialize the pseudotimes. Such trajectories correspond to local modes in the posterior space that are sufficiently narrow the probability of the Gibbs sampler moving to another local mode is negligible. A future extension that would solve this non-identifiability would involve placing priors on the behavior of certain genes across the branches, which combined with more efficient inference would pick out the ‘true’ trajectory.

Please note that an earlier version of this article can be found on bioRxiv (doi: 10.1101/076547).

## Results

### Synthetic datasets

We first demonstrate our method on a synthetic ‘toy’ dataset of 300 bifurcating cells and 60 genes, half of which exhibit differential behavior across the bifurcation and half of which show similar behavior.

Our synthetically generated data is mildly mis-specified with respect to our model to demonstrate robustness when using real genomic data. For example, the generated gene behavior across pseudotime is sigmoidal, which we have previously successfully used to model real single-cell datasets ^11, 12^.

**Figure 2.**
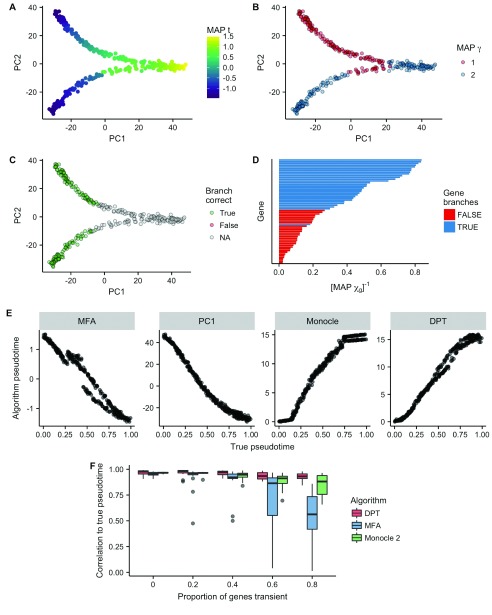
Probabilistic inference of bifurcations in synthetic data. **A** Principal component analysis representation of a toy dataset for 300 cells and 60 genes, colored by the maximum *a posteriori* (MAP) pseudotime estimates. **B** Equivalent representation as ( **A**) color by the MAP branch estimate. **C** Equivalent representation showing whether each branch was assigned correctly. Due to the non-identifiability of mixture components, we map component indices from true to inferred such that the agreement is maximized. **D** The inverse MAP estimates of *χ* largely identify which genes in the dataset exhibit different behavior across the two branches. **E** Comparison of different pseudotime inference algorithms to the ground truth pseudotime on this particular dataset. The algorithms MFA, PC1 (principal component 1), Monocle and DPT had correlations of 0.98, 0.98, 0.98, 0.99 (to 2 s.f.), respectively. **F** The correlation of inferred pseudotimes to ground truth depending on the proportion of genes in the dataset exhibiting transient behavior. MFA shows competitive performance up to around 40% of genes begin transient despite an inherent linear assumption.

Pseudotimes were inferred using Gibbs sampling ( Supplementary File 1) for 10 ^5^ iterations. PCA representations of the synthetic data can be seen in Figures 2A and B, showing the characteristic *Y* shape associated with bifurcating data, colored by both maximum *a posteriori* (MAP) pseudotime and branch assignment estimates, respectively. We compared the Pearson correlation of the estimated pseudotimes to the true pseudotimes ( Figure 2C) for both MFA, PC1 (the first principal component of the data), Monocle and Diffusion Pseudotime, giving values of 0.98, 0.98, 0.98 and 0.99 (to 2 s.f.), respectively. Broad benchmarking of pseudotime algorithms to “ground-truth” data is difficult, due to the inherent assumptions that are necessary about how genes expression evolves along trajectories. However, such toy examples demonstrates the consistency of multiple algorithms on our toy dataset.

One weakness of our model is that it assumes gene expression changes as a linear function of time. This allows us to perform fast conjugate Gibbs sampling, but is highly unrealistic for real data. The synthetic data generated is based on sigmoidal changes across pseudotime, which being nonlinear is already mildly mis-specified with respect to our model. However, genes may also exhibit transient behavior, in which they are briefly down- or up-regulated before returning to their initial state. We sought to quantify the robustness of MFA to transient gene expression by performing extensive simulations. Specifically, we generated synthetic datasets with 0%, 20%,…, 80% of genes exhibiting transient expression, and inferred the pseudotimes using DPT, MFA and Monocle 2. This was repeated 20 times for each percentage of transient genes. The results can be seen in Figure 2F. The performance of MFA remains competitive up to around 40% of genes exhibiting transient expression, after which DPT and Monocle 2 perform significantly better. However, MFA is highly consistent with DPT and Monocle 2 on the two real datasets examined ( Figure 4 and Figure 5), implying the occurrence of transient expression is limited enough in practice for the linearity assumption to be feasible.

One notable difference between MFA and existing bifurcation inference algorithms is in the pre-bifurcation branch assignment. Algorithms, such as Wishbone and DPT, will assign a separate branch to cells preceding the bifurcation. However, MFA will typically assign pre-bifurcation cells to one of the two branches modeled, with the other branch beginning at the bifurcation. A bifurcation process consists of two temporal processes that have a common origin but differing end points. Thus, due to nonidentifiability, cells pre-bifurcation can equally be said to be on one branch with the second beginning at the bifurcation point. Importantly, no matter how we assign the branches under this regime, the observed behavior of genes as a function of both pseudotime and branch assignment will be consistent, which is necessary for biological insight.

### Benefits of modeling zero-inflation

Single-cell RNA-seq data is known to exhibit *dropout*, where a failure to reverse transcribe lowly-expressed mRNA results in zero counts. We have created a variant of MFA that employs an Empirical-Bayes like approach to account for such dropout (see *Methods*). However, a zero count for a particular gene in a particular cell may also be a *true zero* where no mRNA in the cell is present.

**Figure 3.**
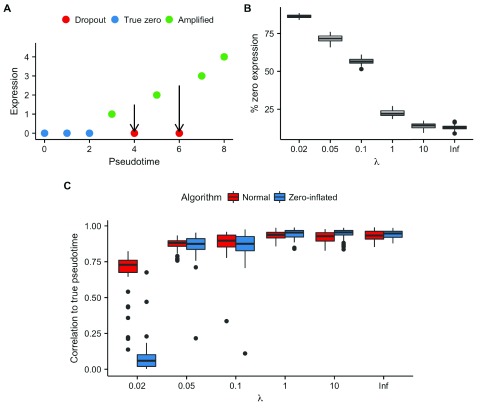
Effects of modeling zero-inflation. **A** Zero counts observed in single-cell RNA-seq data may be attributed to either *true zeros*, where no mRNA of a given gene is produced in a cell, or *dropout*, where there is a failure to reverse-transcribe the low levels of starting material. Alternatively, a count is registered and the gene is *amplified*. In theory not accounting for dropouts will reduce the accuracy of pseudotime inference the two red counts at pseudotimes of 4 and 6 would be ordered with the blue counts. However, in practice it is impossible to distinguish between *dropouts* and *true zeros*. **B** The percentage of counts with zero expression across 50 replicates for each value of *λ* used in dropout simulations. **C** The Pearson correlation to true pseudotime using both the non-zero-inflated and zero-inflated variants of MFA as a function of *λ* used to generate the dataset. Accounting for zero-inflation shows marginal benefits if only a small percentage counts are dropouts. However, for high dropout percentages (> 80%) the algorithm has to “impute” such a large percentage of the data that correlations to the true pseudotime reduce to near-zero.

We expect such true zeros to be useful for pseudotime inference. Figure 3A shows a conceptual model where a gene is up-regulated along pseudotime with two cells exhibiting dropout. The true zeros (in blue) help pseudotime inference as the low-expression implies they are at the beginning of pseudotime. However, the cells exhibiting dropout (in red) would potentially impede pseudotime inference as MFA would order them with the true zero cells at the beginning of the trajectory.

Accounting for such dropouts involves modifying the model so that zero counts are likely if the underlying latent expression is low. Therefore, the red dropout cells in Figure 3A would be effectively imputed (via Gibbs updates) upwards towards the mean expression line, increasing the accuracy of pseudotime inference. However, as there is no way to distinguish between true zeros and dropouts, we also “impute” the expression of the true zeros, which may itself decrease the accuracy of pseudotime inference.

We sought to quantify the benefits of modeling zero inflation against the drawbacks of losing the information contained in “true zeros”. We created multiple synthetic datasets ( Supplementary File 1), while varying the dropout parameter λ ∈ {0.02, 0.05, 0.1, 1, 10, ∞}, where λ = 0.02 has the largest levels of dropout, while λ = ∞ has no dropout, only true zeros. This was repeated 50 times for each *λ*, and the proportion of zero counts in each dataset can be seen in Figure 3B. We subsequently re-inferred the pseudotimes using MFA with both the zero-inflated and standard variants.

The resulting correlations with the true pseudotimes across the range of *λ* and MFA variants can be seen in Figure 3C. At very high levels of dropout ( *λ* = 0.02, where > 80% of counts are zeros) the zero-inflated variant performs considerably worse than the non-zero-inflated variant, with virtually no correspondence to the true pseudotimes compared to *ρ ≈* 0.75. We suggest this is due to the inference procedure, effectively imputing such a large proportion of the data that there are too many degrees of freedom to effectively infer the trajectory. For the remaining values of λ the zero-inflated variant infers pseudotimes largely comparable to those of the non-zero inflated version, with marginal improvements in accuracy when there is moderate dropout ( *λ* = 1, 10). We conclude that incorporating zero-inflation into pseudotime inference is sensible, but the variable quality across the (unknown in practice) dropout range along with considerable additional computational cost render it unnecessary for most practical purposes.

### Application to single-cell RNA-seq data

We next applied our method to previously published single-cell RNA-seq data of 4,423 hematopoietic progenitor/stem cells, differentiating into myeloid and erythroid precursors ^13^.

**Figure 4.**
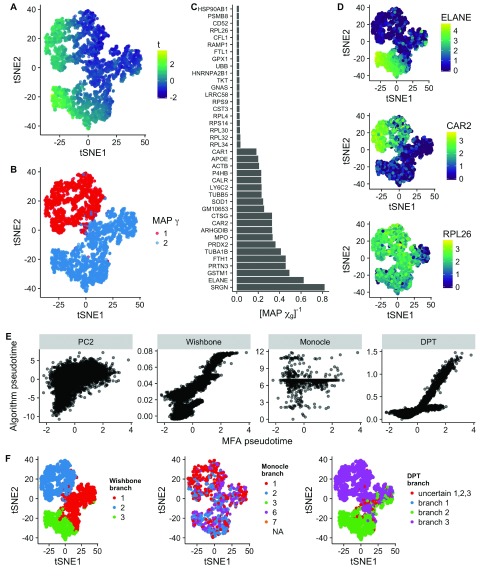
Inference of bifurcations in scRNA-seq data of 4,423 hematopoietic progenitor/stem cells differentiating into myeloid and erythroid precursors ^3^. **A** tSNE representation colored by the maximum *a posteriori* (MAP) pseudotime. **B** Equivalent plot as **A** colored by MAP γ (branch assignment). **C** Inverse map *χ* showing both the 20 largest and 20 smallest values indicating which genes do and do not show differential behavior across the bifurcation. **D** tSNE representation of the dataset colored by gene expression. Both *ELANE* and *CAR2* were predicted by the inverse *χ* values to show differing expression across the branches, while *RPL26* was predicted to show similar expression. **E** Scatter plots of pseudotime values compared to those inferred by PC2, Wishbone, Monocle, and DPT. These had Pearson correlations of 0.54, 0.83, 0.01, and 0.78, respectively. **F** tSNE representations of the dataset colored by branch allocation of alternative algorithms shows good agreement with Wishbone and DPT.

To reduce the dataset to a computationally feasible size we used only genes expressed in at least 20% of cells with a variance in normalized expression greater than 5. We performed Gibbs sampling for 4 *×* 10 ^4^ iterations using default hyperparameter values, except for *τ _θ_* = *τ _η_* = 1, and initialized the pseudotimes to the second principal component of the data. The results can be seen in Figures 4(A and B). The MAP pseudotime estimates clearly recapitulates the trajectory in the data, as shown using a tSNE representation from 3, while the MAP estimates of *γ _i_* detects the branching structure in the data, consistent with previous methods.

We went on to analyze the genes suggested by the model to be involved in the bifurcation process. Figure 4C shows the inverse posterior mean of *χ _g_*, with larger values indicating more evidence that gene *g* is involved in the bifurcation process. For illustration purposes, we plot the expression of *ELANE* and *CAR2*, which the model suggests will show differential behavior across the bifurcation, along with *RPL26*, which the model suggests will show common behavior ( Figure 4D).

We next sought to compare the performance of MFA to existing bifurcation inference algorithms, in particular Wishbone, DPT and Monocle (v2), along with the second principal component of the data (PC2), which we noted from exploratory analyses was highly correlated with the existing Wishbone values. We sub-sampled down to 1,000 cells for Monocle comparisons for computational convenience and used the previously published results for Wishbone (from 3). The root cell for DPT was selected as the cell with the minimum value for the second principal component and similarly the root state for Monocle was chosen such that it contained that cell. Otherwise, algorithms were run with default parameters.

The comparison of the inferred pseudotimes with that MFA can be seen in Figure 4E. There is high correlations with PC2 ( *ρ* = 0.54), Wishbone ( *ρ* = 0.83), and DPT ( *ρ* = 0.78). However, there is virtually no correlation with Monocle ( *ρ* = 0.01), though as this low correlation only occurs with Monocle we assume it is not an issue with MFA. We also sought to compare branch allocations across the algorithms, which is difficult due to the non-identifiability of the statistical models involved. Figure 4F shows a tSNE representation of the cells colored by branch allocation for each of Wishbone, Monocle and DPT. We see that MFA is largely consistent with Wishbone and DPT, detecting a bifurcation at the “pinch” in the tSNE plot, but as with the pseudotimes there is barely any correspondence in branch allocations with Monocle (which, as of version 2, does not allow pre-specification of the number of branches to model).

### Application to single-cell mass-cytometry data

We next applied MFA to single-cell mass cytometry data, tracking the differentiation of 22,850 monocytes and erythrocytes from hematopoietic stem and progenitor cells across 12 markers as published in 14 and previously analyzed in 3. For computational convenience with all algorithms, we sub-sampled the data down to 2,000 randomly chosen cells, with the exception of Monocle, which we subsequently sub-sampled further down to 1,000 cells. We found that due to the small number of proteins measured there was too much freedom for the MFA model to infer mixtures using the default parameter settings. We therefore had to encourage large levels of similarity across the two branches by setting *α _χ_* = 5 *×* 10 ^3^ and *β _χ_* = 1.

**Figure 5.**
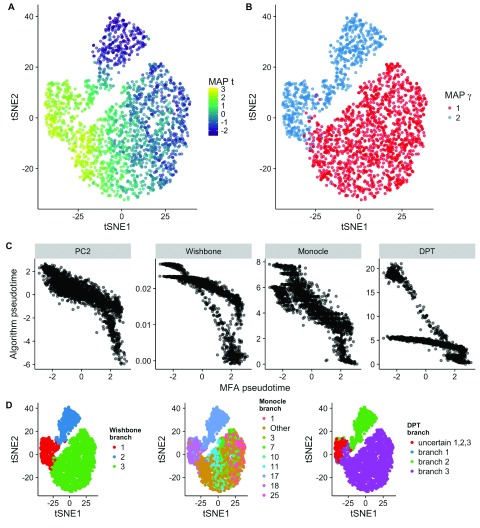
Inference of bifurcations in single-cell mass cytometry data of a subsample of 2,000 hematopoietic progenitor/stem cells differentiating into monocyte and erythrocyte progenitors ^3^. **A** A tSNE representation colored by the maximum *a posteriori* (MAP) pseudotime. **B** Equivalent plot as **A** colored by MAP *γ* (branch assignment). **C** Scatter plots of MFA pseudotime compared to PC2, Wishbone, Monocle, and DPT, with Pearson correlations of 0.84, 0.86, 0.80 and 0.69 respectively. **D** tSNE representation colored by branch assignment of Wishbone, Monocle, and DPT. As of version 2, Monocle does not allow for the number of branches to be selected *a priori* and typically returns a large number. For the convenience of visualization we therefore only display the 30% most frequent states and group the remaining infrequent ones into “Other”. The figures suggest a good agreement of branch assignment of MFA with Wishbone and DPT, and moderate agreement with Monocle.

The results can be seen in Figure 5. Figure 5A shows a tSNE representation (as published in 3) showing the inferred MAP pseudotimes correctly following the left-right trajectory, while Figure 5B correctly shows the MAP *γ* values identifying a bifurcation at the “pinch” in the plot.

We subsequently compared the inferred pseudotimes and branching to those found using the alternative algorithms. We found good correspondence to all other methods ( Figure 5C), with Pearson correlations of 0.84, 0.86, 0.80 and 0.69 for PC2, Wishbone, Monocle, and DPT, respectively. We further compared the branch assignment of MFA to those of the alternative algorithms ( Figure 5D). As of version 2, Monocle does not allow for the number of branches to be selected *a priori* and typically returns a large number. For the convenience of visualization we therefore only display the 30% most frequent states and group the remaining infrequent ones into “Other”. We find good agreement between MFA and Monocle and DPT, and similarities with the Monocle assignments (MFA branch 2 loosely corresponds to Monocle branch 17).

## Discussion

In this paper we have presented a Bayesian hierarchical mixture of factor analyzers for inference of bifurcating trajectories in single-cell data. Our model is unique compared to existing efforts in that it (a) is fully generative, incorporating measurement noise into inference, (b) jointly infers both the pseudotimes and branches compared to post-hoc inference of branch detection, and (c) jointly infers which genes are differentially regulated across the branches. We also proposed an extension that accounts for the high levels of zero-inflation present in single-cell RNA-seq data. We applied our model to a range of synthetic and real datasets and demonstrated it performs competitively with existing methods.

There is a natural trade-off in designing such models between flexibility and practicality. The implicit assumption of MFA that gene expression develops linearly across pseudotime allows for fast Markov-Chain Monte Carlo sampling and joint inference of branch structure. However, it is potentially highly mis-specified: the predicted expression can become negative leading to erroneous inference (see Supplementary File 1). A solution to this would be to not explicitly assume a strongly parametric form of gene expression and consider nonparametric methods. However, such methods are often overly flexible, requiring either additional capture information to correctly infer pseudotimes ^15^ or hard-setting the pseudotimes prior to inferring the branching structure ^16^. As such there is a natural trade-off between the expressivity of such models and being able to perform valid statistical inference that fully incorporates parameter variation without additional constraints or “tweaking”.

There are several extensions that can be applied to our model. While the model performs well on large single cell RNA-seq datasets, it could be scaled up further using Stochastic Variational Inference ^17^, which due to the model’s conditionally conjugate structure could be implemented without resorting to approximations. As mentioned previously, one main weakness of the model is the unrealistic assumption of linear changes in expression over pseudotime, leading to severe model specification. One could therefore consider alternative nonlinear functions, such as sigmoids (previously used in 8), or nonparametric models such as Gaussian Process Latent Variable Models (previously used in 15, 18), with appropriate structural constraints.

## Software availability

MFA software available from: http://www.github.com/kieranrcampbell/mfa

Archived source code as at time of publication: doi, 10.5281/zenodo.345981 ^9^

License: GNU General Public License (GPL)

## Supplementary material

**Supplementary File 1: Further methods and analysis.** This file contains (1) additional technical details of the statistical inference methods, (2) further description of the zero-inflation, (3) details of the simulation strategy and (4) a discussion of the limitations of the linear model.
