# Supplementary material for: Probabilistic modeling of bifurcations in single-cell gene expression data using a Bayesian mixture of factor analyzers
Source: Wellcome Open Res. 2017 Mar 15;2:19. [Version 1] doi: 10.12688/wellcomeopenres.11087.1 (PMC5428745; doi:10.12688/wellcomeopenres.11087.1)
Supplement: Supplementary file 1 [file wellcomeopenres-2-11959-s0000.tgz › 1cb4b727-ab36-4cc0-9159-f0455e8e1e9e.pdf]

Supplementary File 1
Further methods and analysis

Kieran R Campbell & Christopher Yau

March 6, 2017

Contents

1 Gibbs updates and inference **2**
1.1 Update for γ . . . 2
1.2 Update for k . . . 2
1.3 Update for c . . . 2
1.4 Update for ω . . . 2
1.5 Update for t . . . 3
1.6 Update for $\boldsymbol{\tau}$ . . . 3
1.7 Update for η . . . 3
1.8 Update for θ . . . 3
1.9 Update for χ . . . 3
1.10 Validation of updates . . . 3

2 Modelling zero inflation **4**

3 Generation of synthetic datasets **4**

4 Limits of linear latent variable models **7**

1 Gibbs updates and inference

The full model is specified by

$$
\begin{aligned}
\boldsymbol{\omega} &\sim \text{Dirichlet}(1/B, \ldots, 1/B) \\
\gamma_i &\sim \text{Categorical}(\boldsymbol{\omega}) \\
\eta &\sim \text{Normal}(\tilde{\eta}, \tau_\eta^{-1}) \\
\theta_g &\sim \text{Normal}(\tilde{\theta}, \tau_\theta^{-1}) \\
\chi_g &\sim \text{Gamma}(\alpha_\chi, \beta_\chi) \\
\mathbf{c}_{\gamma_i} &\sim \text{Normal}(\eta_{\gamma_i}, \tau_c^{-1}) \\
\mathbf{k}_{\gamma_i} &\sim \text{Normal}(\boldsymbol{\theta}, \boldsymbol{\chi}^{-1}\mathbb{1}_G) \\
t_i &\sim \text{Normal}(0, 1) \\
\boldsymbol{\tau} &\sim \text{Gamma}(\alpha, \beta) \\
\mathbf{y}_i &\sim \text{Normal}(\mathbf{c}_{\gamma_i} + \mathbf{k}_{\gamma_i} t_i, \boldsymbol{\tau}^{-1}\mathbb{1}_G)
\end{aligned} \tag{1}
$$

where $\mathbf{y}_i$ is a G-length vector of expression in cell i, B is the number of branches modelled and we define $\Lambda_\gamma = [\mathbf{c}_\gamma \; \mathbf{k}_\gamma]$, $\gamma \in 1, \ldots, B$.

1.1 Update for γ

Defining $\pi_{i,b} = p(\gamma_i = b)$ (the probability that cell i is on branch b) then

$$p(\gamma_i | \mathbf{y}_i, t_i, \{\Lambda_b\}_{b=1}^B, \boldsymbol{\omega}, \boldsymbol{\tau}) = \text{Categorical}(\boldsymbol{\pi}_i) \tag{2}$$

where

$$\pi_{i,b} = \frac{\omega_b \text{Normal}(\mathbf{y}_i | \mathbf{c}_b + \mathbf{k}_b t_i, \boldsymbol{\tau}^{-1})}{\sum_{b'=1}^B \omega_{b'} \text{Normal}(\mathbf{y}_i | \mathbf{c}_{b'} + \mathbf{k}_{b'} t_i, \boldsymbol{\tau}^{-1})} \tag{3}$$

1.2 Update for k

$$k_{gb} | \boldsymbol{\gamma}, c_{gb}, \mathbf{Y}, \boldsymbol{\tau}, \mathbf{t} \sim \text{Normal}(\nu_{gb}^k, 1/\lambda_{gb}^k) \tag{4}$$

where

$$
\begin{aligned}
\nu_{gb}^k &= \frac{\tau_g \sum_{i:\gamma_i=b} t_i (y_{ig} - c_{gb})}{\tau_k + \tau_g \sum_{i:\gamma_i=b} t_i^2} \\
\lambda_{gb}^k &= \tau_k + \tau_g \sum_{i:\gamma_i=b} t_i^2
\end{aligned} \tag{5}
$$

1.3 Update for c

$$c_{gb} | \boldsymbol{\gamma}, k_{gb}, \mathbf{Y}, \boldsymbol{\tau}, \mathbf{t} \sim \text{Normal}(\nu_{gb}^c, 1/\lambda_{gb}^c) \tag{6}$$

where

$$
\begin{aligned}
\nu_{gb}^c &= \frac{\tau_g \sum_{i:\gamma_i=b} (y_{ig} - k_{gb} t_i)}{\tau_c + N_b \tau_g} \\
\lambda_{gb}^c &= \tau_c + N_b \tau_g
\end{aligned} \tag{7}
$$

and N_b is the number of cells assigned to branch b at that iteration.

1.4 Update for ω

$$\boldsymbol{\omega} | \{N_b\}_{b=1}^B, B \sim \text{Dirichlet}(1/B + N_1, \ldots, 1/B + N_B) \tag{8}$$

1.5 Update for t

$$t_i|\boldsymbol{\gamma}, \{\Lambda_b\}_{b=1}^B, \boldsymbol{\tau} \sim \text{Normal}(\nu_i^t, 1/\lambda_i^t) \tag{9}$$

where

$$\begin{aligned} \nu_i^t &= \frac{\sum_g \tau_g k_{g\gamma_i}(y_{ig} - c_{g\gamma_i})}{1 + \sum_g \tau_g k_{g\gamma_i}^2} \\ \lambda_i^t &= 1 + \sum_g \tau_g k_{g\gamma_i}^2 \end{aligned} \tag{10}$$

1.6 Update for $\boldsymbol{\tau}$

$$\tau_g|\{\Lambda_b\}_{b=1}^B, \mathbf{t}, \boldsymbol{\gamma} \sim \text{Gamma}\left(\alpha + N/2, \beta + \sum_{i=1}^N \frac{(y_{ig} - \mu_{ig})^2}{2}\right) \tag{11}$$

where $\mu_{ig} = c_{g\gamma_i} + k_{g\gamma_i} t_i$.

1.7 Update for η

$$\eta|\tau_c, \{\mathbf{c}_b\}_{b=1}^B, \tau_\eta, \tilde{\eta} \sim \text{Normal}(\nu^\eta, 1/\lambda^\eta) \tag{12}$$

where

$$\begin{aligned} \nu^\eta &= \frac{\tau_c \sum_{b,g} c_{gb} + \tau_\eta \tilde{\eta}}{BG\tau_c + \tau_\eta} \\ \lambda^\eta &= BG\tau_c + \tau_\eta \end{aligned} \tag{13}$$

1.8 Update for θ

$$\theta_g|\boldsymbol{\chi}, \{\mathbf{k}_b\}_{b=1}^B, \tau_\theta, \tilde{\theta} \sim \text{Normal}(\nu_g^\theta, 1/\lambda_g^\eta) \tag{14}$$

where

$$\begin{aligned} \nu_g^\theta &= \frac{\chi_g \sum_b k_{gb} + \tau_\theta \tilde{\theta}}{B\chi_g + \tau_\theta} \\ \lambda_g^\theta &= B\chi_g + \tau_\theta \end{aligned} \tag{15}$$

1.9 Update for χ

$$\chi_g|\{\mathbf{k}_b\}_{b=1}^B, \theta_g, \alpha_\chi, \beta_\chi \sim \text{Gamma}\left(\alpha_\chi + \frac{B}{2}, \beta_\chi + \frac{\sum_b (k_{gb} - \theta_g)^2}{2}\right) \tag{16}$$

1.10 Validation of updates

All Gibbs updates were checked numerically using the Gwecke test (see e.g. [1]). This exploits the identity

$$\frac{p(\theta = x|\Theta, \mathcal{D})}{p(\theta = x'|\Theta, \mathcal{D})} = \frac{p(\theta = x, \Theta|\mathcal{D})}{p(\theta = x', \Theta|\mathcal{D})} \tag{17}$$

which should hold up to the numerical precision of the computer used. We can therefore use the function that computes the likelihood (which we have implemented to monitor convergence) to ensure the conditional updates are correct, and vice versa.

2 Modelling zero inflation

Single-cell RNA-seq data is known to exhibit *dropout*, where lowly expressed genes register as zero counts. Several computational methods attempt to correct for this. For example, ZIFA [2] proposes a double-exponential dropout model where $p(\text{dropout}) \propto \exp(-\lambda x^2)$ and x is the latent expression. However, the difficulty here is that we must sample from the conditional distribution $p(\lambda|\cdot)$, which to the best of our knowledge does not exist analytically.

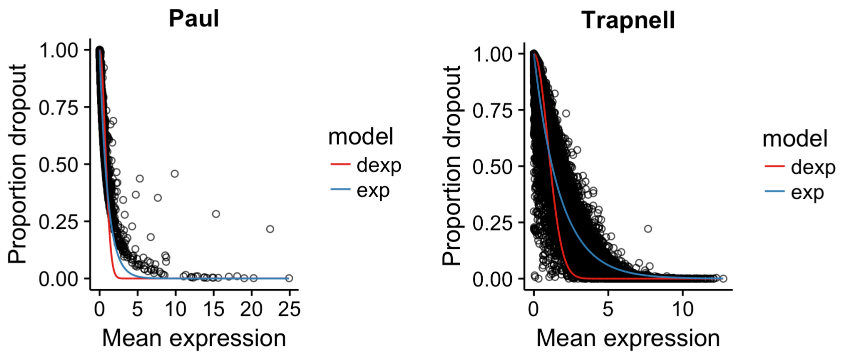

Figure 1: Dropout relationships in single-cell RNA-seq data.

As a solution to this we propose an Empirical-Bayes like procedure to estimate λ globally then infer the latent expression x through further Gibbs updates. First we note that a single exponential dropout empirically fits the dropout relations in single-cell RNA-seq datasets better than the double exponential dropout (figure 1). We subsequently modify the likelihood to give a per-gene dropout probability of $p(\text{dropout in gene } g) = \exp(-\lambda \sum_{i=1}^{N} x_{ig})$ which depends on the mean latent expression level of the gene. While this is of course an approximation and we expect the probability of a dropout to be specific to each gene in each cell depending on the latent expression, this allows us to estimate λ by fitting the maximum likelihood exponential curve of the proportion of cells a gene is expressed in against the mean expression level (similar to figure 1) using the `R` function `nls`. Thus the modified likelihood becomes

$$\begin{aligned} \mathbf{x}_i &\sim \text{Normal}(\mathbf{c}_{\gamma_i} + \mathbf{k}_{\gamma_i} t_i, \boldsymbol{\tau}^{-1} \mathbb{1}_G) \\ h_{ig} &\sim \text{Bernoulli}(\exp\left(-\frac{\lambda}{N} \sum_{i'} x_{i'g}\right)) \\ y_{ig} &= \begin{cases} x_{ig} & \text{if } h_{ig} = 0 \\ 0 & \text{if } h_{ig} = 1 \end{cases} \end{aligned} \tag{18}$$

Note that since h_{ig} is effectively observed we only need to Gibbs sample x_{ig} for which $h_{ig} = 1$. The conditional distribution for x_{ig} is then given by

$$x_{ig}|\cdot \sim \text{Normal}\left(\mu_{ig} - \frac{\lambda}{N\tau_g}, \tau_g^{-1}\right) \tag{19}$$

where μ_{ig} is defined as above.

3 Generation of synthetic datasets

Synthetic datasets were generated for various simulations throughout the analysis. Rather than simply generating data from the model to reinfer, we attempted to create synthetic data that was as close to real single-cell data as feasible, meaning *the synthetic data is severely misspecified with respect to our model.* The full details can be found in algorithm 1; we describe various design choices below.

The first consideration is the functional form of gene expression along pseudotime. A linear assumption is fundamentally unrealistic as the gene expression cannot go to $\pm\infty$ as pseudotime progresses. Consequently we adopt sigmoidal expression across pseudotime (previously suggested in [3, 4]), parameterised by the half-peak expression ϕ, the *switch-time* δ and the switch-strength k:

$$\text{Sigmoid}(t, k, \phi, \delta) = \frac{2\phi}{1 + \exp(-k(t - \delta))} \tag{20}$$

For half of the G genes we assume the expression along the two branches is the same, thus we model common k, ϕ and δ parameters. For the second half we assume the expression diverges, and in particular for each gene $k = 0$ for one of the branches, which we can call b_0, and b_1 for the branch for which $k \neq 0$. If k on the other branch is positive (ie $k_{gb_1} > 0$) then we set the half-peak expression on b_0 to zero, as the genes must both start at 0, and if one turns on the other must remain off (figure 2A). Alternatively, if $k_{gb_1} < 0$ then the genes must begin in an *on* state and switch off for cells on b_1 (figure 2B). Thus we set ϕ_{gb_0} to twice its original value. For any gene that shows divergent behaviour across branches we set δ to be in the second half of the trajectory. We then construct the mean, using the sigmoid function, and generate the data from a Gaussian noise model ensuring any negative values are set to zero. This gives the characteristic bifurcation pattern in PCA space as seen in figures 2E&F.

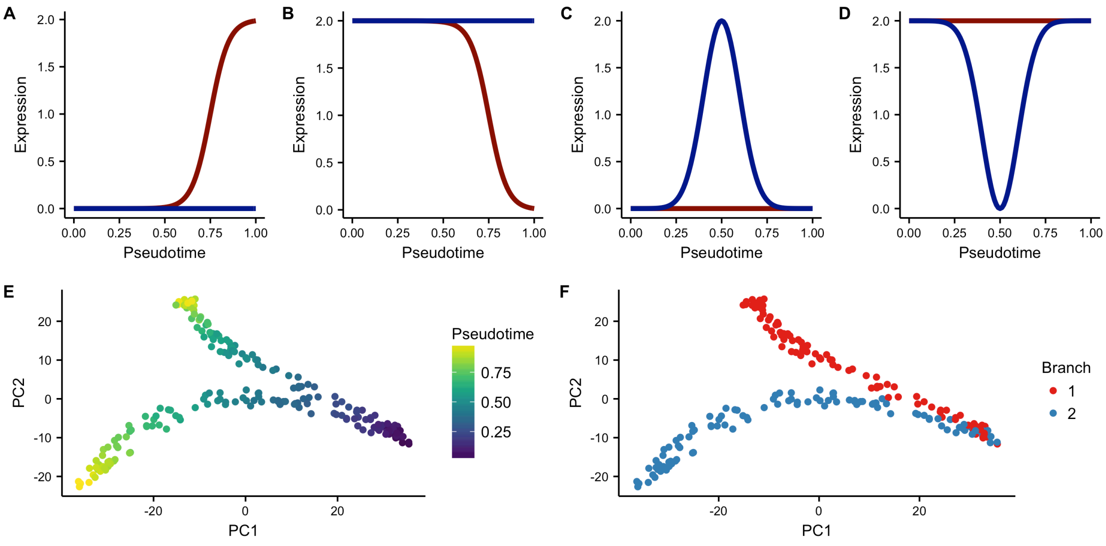

Figure 2: Generation of synthetic data

We may also wish to generate transiently expressed genes to test the limits of the monotonicity assumptions in our model. Transient behaviour can either be across both branches or exhibit divergent behaviour (transient on one branch only). To simulate transient genes we swap out the sigmoidal mean function for a Gaussian function centred around the mid-point of the trajectory:

$$\text{Transient}(t, l, s) = \exp(-\frac{1}{2s}(t - l)^2) \tag{21}$$

We additionally ensure the behaviour is constrained to be identical on each branch at the beginning and end of the trajectory. Examples of such behaviour may be seen in figures 2C&D.

If we would like to incorporate zero-inflation, we calculate a dropout probability for each measurement via $p_{ig} = \exp(-\lambda x_{ig})$, where λ is set to a reasonable value based on observations of real datasets. Note that our model is mis-specified with respect to this as it models a per-gene dropout probability p_g.

Algorithm 1 Generate pseudotemporally regulated bifurcating scRNA-seq data

Data: G genes, C cells, proportion of genes exhibiting transient expression $p_{\text{transient}}$
Result: A $C \times G$ matrix of gene expression X, where $[X]_{cg} = x_{cg}$
for $g \in 1 \dots G$ **do**
    Sample $\sigma_g^2 \sim$ Gamma(2,2)
    for $b \in \{1, 2\}$ **do**
        Draw $k_{gb} \sim$ Unif(5, 10)
        Draw $\phi_{g\cdot} \sim$ Unif(5,10)
        Draw $\delta_{g\cdot} \sim$ Unif(a, b) where $a = 0, b = 0.5$ if $g < G/2$ and $a = 0.5, b = 1$ if $G \geq G/2$
        Set $k_{bg} \leftarrow -k_{bg}$ with probability $\frac{1}{2}$
    end for
end for
for $g \in G/2, \dots, G$ **do**
    Set $b \leftarrow 1$ or 2 each with probability $\frac{1}{2}$
    Set $k_{gb} \leftarrow 0$
    Set $b_0 = b' : k_{gb'} = 0, b_1 = b' : k_{gb'} \neq 0$
    if $k_{b_1 g} > 0$ **then**
        $\phi_{gb_0} \leftarrow 0$
    else
        $\phi_{gb_0} \leftarrow 2\phi_{gb_1}$
    end if
end for
for $c \in 1, \dots, C$ **do**
    Sample $t_c \sim$ Unif(0,1)
    Sample γ_c from $\{1, 2\}$ each with probability $\frac{1}{2}$
    for $g \in 1, \dots, G$ **do**
        Set $\mu \leftarrow \text{Sigmoid}(t_c, k_{g\gamma_c}, \phi_{g\gamma_c}, \delta_{g\gamma_c})$
        Sample $x_{cg} \sim \text{N}(\mu, \sigma_g^2)$
    end for
end for
Set T as the set of randomly sampled indicies such that $|T| \approx p_{\text{transient}} G$
for $t \in T$ **do**
    if $t < G/2$ **then**
        Sample $s_t \sim \text{LogNormal}(\log(0.05), 0.5)$
        for $c \in 1, \dots, C$ **do**
            $\mu \leftarrow \text{Transient}(t_c, 0.5, s_t)$
            $\mu \leftarrow 1 - \mu$ with probability $\frac{1}{2}$
            Sample $x_{ct} \sim \text{Normal}(2\phi_{t1}\mu, \sigma_t^2)$
        end for
    else
        Set $b_0 = b' : k_{tb'} = 0, b_1 = b' : k_{tb'} \neq 0$
        Sample $s_t \sim \text{LogNormal}(\log(0.05), 0.3)$
        for $c \in 1, \dots, C$ **do**
            $\mu \leftarrow \text{Transient}(t_c, 0.75, s_t)$
            if $k_{tb_1} < 0$ **then**
                $\mu \leftarrow 1 - \mu$
            end if
            if $\gamma_c = b_1$ **then**
                Sample $x_{ct} \sim \text{Normal}(\mu, \sigma_t^2)$
            end if
        end for
    end if
end for
for $g \in 1, \dots, G,\ c \in 1, \dots, C$ **do**
    if $x_{cg} < 0$ **then**
        Set $x_{cg} \leftarrow 0$
    end if
end for

4 Limits of linear latent variable models

In general we expect linear latent variables to be highly mis-specificed with respect to real gene expression data - gene expression will rarely evolve linearly as a function of time, nor even necessarily monotonically. A surprising result is that such an assumption is in practice sufficient to recapitulate the results on real data of algorithms that specifically account for nonlinearities in the data. We can therefore assume that the majority of genes in real datasets behave approximately linearly.

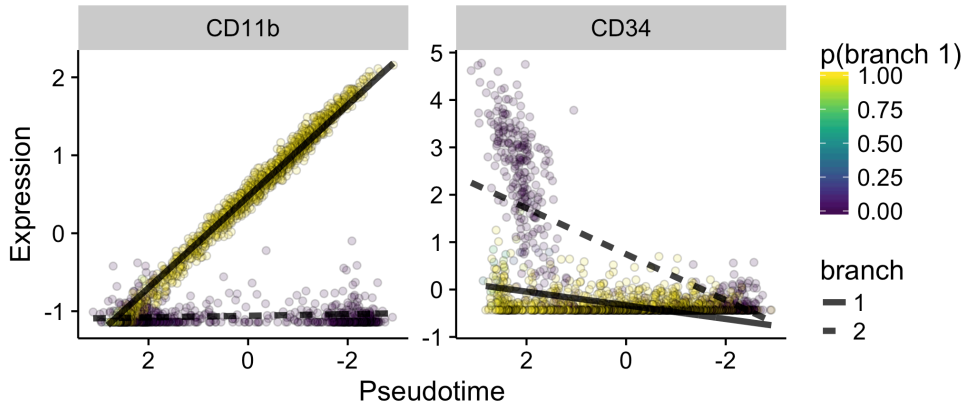

Figure 3:

Such mis-specification - though conduisive to fast full MCMC inference - does come at a cost. An example is given in figure 3 of the mass cytometry data presented in the main text (we have reversed the pseudotimes so time runs from left to right). The gene *CD11b* displays differential regulation across the branches - up-regulation on branch 1 and constant expression on branch 2. The model fits two values of k for this as can be seen by the black line fits, and this the value of χ is sufficiently small that we correctly designate it as a gene that bifurcates.

However, the gene *CD34* is incorrectly designated as one that should bifurcate when in fact it doesn't. Due to model mis-specification the cells with non-zero expression at the beginning are "hard-assigned" to branch 2, when in fact they should be equally assigned to both branches 1 & 2. Consequently, $|k_2| \gg |k_1|$ and the value of χ indicates that the gene is involved in the bifurcation, when in fact it isn't. Such incorrect inferences can easily be checked visually on a reduced-dimension representation.

References

[1] Roger B Grosse and David K Duvenaud. Testing mcmc code. *arXiv preprint arXiv:1412.5218*, 2014.

[2] Emma Pierson and Christopher Yau. Zifa: Dimensionality reduction for zero-inflated single-cell gene expression analysis. *Genome biology*, 16(1):1, 2015.

[3] Kieran R Campbell and Christopher Yau. Order under uncertainty: robust differential expression analysis using probabilistic models for pseudotime inference. *PLOS Computational Biology*, 12(11):e1005212, 2016.

[4] Kieran R Campbell and Christopher Yau. switchde: Inference of switch-like differential expression along single-cell trajectories. *Bioinformatics*, page btw798, 2016.
