# Supplementary material for: Probabilistic modeling of bifurcations in single-cell gene expression data using a Bayesian mixture of factor analyzers
Source: Wellcome Open Res. 2017 Mar 15;2:19. [Version 1] doi: 10.12688/wellcomeopenres.11087.1 (PMC5428745; doi:10.12688/wellcomeopenres.11087.1)
Supplement: Peer review history

# Peer review: Referee response for version 1

**DOI:** 10.21956/wellcomeopenres.11959.r21991

**Published:** 2017-05-09

**Authors:** Luca Pinello

**Affiliations:** 1 Department of Pathology, Massachusetts General Hospital and Harvard Medical School, Boston, MA, USA

Campbell and Yau present a probabilistic method called MFA to infer bifurcating trajectories and differentially regulated genes across branches from single cell transcriptomic data.

 The method is based on a Bayesian hierarchical mixture of factor analyzers. The authors also discuss an extension of this method to deal with dropout events commonly observed in scRNA-seq datasets. Although they claim that the model obtained with this procedure is less misspecified, the computational requirements associated with it may be a burden, especially for very sparse gene expression matrices and unnecessary for practical purposes.

 MFA is evaluated on a synthetic dataset, scRNA-seq and mass cytometry data and compared with existing methods, although limited to Wishbone, Monocle2 and Diffusion Pseudotime.

 Overall, the manuscript is well written, the results clearly described and the source code provided.

 It may be helpful to consider/clarify the following points to improve the manuscript:

1. The computational requirements of MFA are not discussed in depth and running times are not reported. Please add a table comparing the execution times of the different methods tested using the full datasets presented and not the down-sampled versions. If a method fails to run in reasonable time, just report this fact (and if possible show the speed-up of MFA using down-sampled datasets). 
2. For different datasets, different values of (hyper)parameters are used but no clear guidelines are provided to the user in how to set those values. Please explain the rationale and if possible clear metrics that the users can use for tuning those parameters. 
3. *“PCA is applied to the data before inference and the principal component that best corresponds to the trajectory based on the expression of known genes is used to initialize the pseudotimes”.* Please explain how to initialize the pseudo-time in absence of known genes. 
4. I agree with Dr. Gitter about adding a short intro to factor analysis before jumping to equation 2. 
5. Please describe (or better show with a synthetic dataset) how MFA performs when more than one branching point is present. For example, using the synthetic dataset presented in Rizvi et al 2017 Nat Biotech (see Figure 2). 
6. The authors claim that explicitly modeling the dropout events doesn’t always justify the computational cost. I think it may be worth to test this idea on real datasets, especially droplet based (drop-seq, in-drop or 10x genomics) in which this problem is more pronounced. Good candidate datasets to show how the method performs in those settings are presented in van Dijk et al. ^1^ where their imputation strategy clearly improve pseudotime estimations or Zheng et al. 2016 Nat Comm. 
7. Application to scRNA-seq: 
  1) What is the running time without down-sampling? How comparable are the results with or without down-sampling?
  2) Two genes (ELANE and CAR2) are presented to illustrate the bifurcation process, what other genes are significant by this analysis? It may be worth to show in a sup table the ranking obtained for each branch using MFA  (are *ELANE and CAR2 on top?*).
  3) *“The comparison of the inferred pseudotimes with that MFA can be seen in Figure 4E. There is high correlations with PC2 (* *ρ* *= 0.54), Wishbone (* *ρ* *= 0.83), and DPT (* *ρ* *= 0.78).* *”* Please describe more explicitly how the correlation is calculated taking into account the fact that different approaches may have different number of branches (and that some genes may be relevant only in a sub-branch).
8. Application to mass-cytometry data:
  1) Please report the results and running time using the whole dataset. If a method fails to run in a reasonable time exclude it from the comparison.
  2) Custom parameters used (see point 2)
9. In the plot where multiple cells are displayed as circles, it may be worth to remove the black border to improve the perception of the density (for example Figure 2e or Figure 4e).

I have read this submission. I believe that I have an appropriate level of expertise to confirm that it is of an acceptable scientific standard.

## References

[1] van DijkDNainysJSharmaRKathailPCarrAJMoonKRMazutisLWolfGKrishnaswamySPe'erD: MAGIC: A diffusion-based imputation method reveals gene-gene interactions in single-cell RNA-sequencing data. *BioRxiv*.2017;

# Peer review: Referee response for version 1

**DOI:** 10.21956/wellcomeopenres.11959.r21016

**Published:** 2017-04-06

**Authors:** Anthony Gitter

**Affiliations:** 1 Department of Biostatistics and Medical Informatics, University of Wisconsin–Madison, Madison, WI, USA

This manuscript presents Mixtures of Factor Analysers (MFA), a hierarchical Bayesian model for studying the branching structure in single-cell RNA-seq and mass cytometry data.  Single-cell RNA-seq can provide snapshots of cells progressing through dynamic biological processes, many of which exhibit branching structure in which the expression levels of one subset of cells diverges from the others.  These types of dynamic behaviors are encountered not only in differentiation but also in stimulus response and other processes, which has sparked a need to computationally model the overall branching structure of the process, how cells progress through the process, and how gene expression levels of some genes differ along the branches.  These and related inference problems are what MFA aims to solve.

 MFA is a generative probabilistic model that uses factor analysis to model the expression properties of branches in a single-cell RNA-seq dataset.  A prior is used to encourage similar factor loading gradients in each branch.  An optional extension models the dropout phenomenon in which technical artifacts can cause non-zero mRNA abundances to be reported as zeros.  MFA is compared with several popular existing algorithms that are not generative probabilistic models: Wishbone, Diffusion Pseudotime, and Monocle 2.  These comparisons are conducted in a fair manner on simulated and real data.  The assessment of the benchmarking is balanced, as is the overall conclusion that in most cases MFA is competitive with these existing approaches even if there is not evidence that it definitively outperforms them by some quantitative metric.

 The balanced discussion is a strength of the manuscript overall.  Figures 2E and 3C both show the scenarios in which MFA's performance degrades with respect to dropout levels or the fraction of transient genes.  The authors conclude that in many practical analyses the extension for incorporating zero-inflation is not worth the added computational cost.  They also present the limitations of their linear model and offer suggestions for improving the scalability of the inference and the linearity assumption.

 The open source software is another asset and follows the best practices for scientific code.  The code is available in GitHub, and an archival version has been deposited in Zenodo.  The Zenodo version's title states that it is the "Bioconductor-ready version", and providing the mfa R package through Bioconductor would indeed further enhance its utility.

 Overall, the manuscript is easy to read, and the model is technically sound and well-motivated.  I have only minor comments that may improve the accessibility to a broader audience and help clarify some points.

 Minor comments:

 * The Methods section assumes that the reader is already familiar with factor analysis, as this technique is not explained.  It would be helpful to introduce the approach and the meaning of c and k in this biological context.

 * There has been other related work on branching trajectories in single-cell datasets.  A few examples include:

- SLICER (DOI:10.1186/s13059-016-0975-3) ^1^
- TSCAN (DOI:10.1093/nar/gkw430) ^2^
- Topslam (DOI:10.1101/057778) ^3^
- Mpath (DOI:10.1038/ncomms11988) ^4^

Very briefly discussing some of these methods and expanding the discussion of how GPfates (reference 16, DOI:10.1126/sciimmunol.aal2192) ^5^ relates to MFA would help readers understand MFA's advantages and disadvantages.  I do not think it is necessary to benchmark against additional algorithms.

 * The parameter B, the number of branches, appears to be user-defined, but this is not explicitly stated in the text.  It would help users to offer guidance on selecting this crucial parameter.

 * The sensitivity to the hyperparameter values is not assessed.  It is not clear what model behavior was observed when modeling the mass cytometry dataset that led to the decision to use non-default values for alpha_x and beta_x and how users should make those decisions on new datasets.

 * I understand the mathematical invariance presented in Figure 1, but the biological argument is not intuitive.  In the bifurcation 2 -> 1,3 states 1 and 3 have the same expression level, which would suggest that this single gene does not exhibit branching behavior.  Rather, it switches from a high to low state in all cases.

 * The simulation with 300 bifurcating cells and 60 genes may have been too simple.  Even the first principal component of the data recovers the true pseudotimes well.  All of the methods perform extremely well, making it difficult to assess their relative performances.

 * I expected that the red cells along the lower curve in Figure 2B would be labeled as False in Figure 2C.  Visually, the branch point appears to occur around 15 on the PC1 axis.

 * Running Monocle 2 on a smaller set of sub-sampled cells than the other methods could put it at a disadvantage.  The scalability of MFA is not discussed or related to the runtimes of the other methods.  Could MFA run on the full mass cytometry dataset in a reasonable amount of time or is sub-sampling required?

 * There are a few potential typos:

- Abstract: "apply or model" -> "apply our model"
- Abstract: "context practical" -> "context of practical"
- Supplement page 2: The identity matrix symbol in the line of Equation 1 for k is not correct
- Supplement page 4: Presumably the p(dropout in gene g) equation in the text should also have a 1/N term to match Equation 18
- Supplement page 7: "conduisive" -> "conducive"

I have read this submission. I believe that I have an appropriate level of expertise to confirm that it is of an acceptable scientific standard.

## References

[1] WelchJDHarteminkAJPrinsJF: SLICER: inferring branched, nonlinear cellular trajectories from single cell RNA-seq data. *Genome Biol*.2016;17(1) : 10.1186/s13059-016-0975-3 106 10.1186/s13059-016-0975-3 27215581PMC4877799

[2] JiZJiH: TSCAN: Pseudo-time reconstruction and evaluation in single-cell RNA-seq analysis. *Nucleic Acids Res*.2016;44(13) : 10.1093/nar/gkw430 e117 10.1093/nar/gkw430 27179027PMC4994863

[3] ZwiesseleMLawrenceND: Topslam: Waddington Landscape Recovery for Single Cell Experiments. *bioRxiv*.2017; 10.1101/057778 10.1101/057778 Reference source

[4] ChenJSchlitzerAChakarovSGinhouxFPoidingerM: Mpath maps multi-branching single-cell trajectories revealing progenitor cell progression during development. *Nat Commun*.2016;7: 10.1038/ncomms11988 11988 10.1038/ncomms11988 27356503PMC4931327

[5] LönnbergTSvenssonVJamesKRFernandez-RuizDSebinaIMontandonRSoonMSFoggLGNairASLiligetoUStubbingtonMJLyLHBaggerFOZwiesseleMLawrenceNDSouza-Fonseca-GuimaraesFBunnPTEngwerdaCRHeathWRBillkerOStegleOHaqueATeichmannSA: Single-cell RNA-seq and computational analysis using temporal mixture modelling resolves Th1/Tfh fate bifurcation in malaria. *Sci Immunol*.2017;2(9) : 10.1126/sciimmunol.aal2192 10.1126/sciimmunol.aal2192 28345074PMC5365145
